# Immunoproteomic approach identifies a putative virulence chaperone DnaK protein as a candidate diagnostic marker and therapeutic target for *Pythium insidiosum* infection

**DOI:** 10.1016/j.heliyon.2025.e42487

**Published:** 2025-02-06

**Authors:** Chalisa Jaturapaktrarak, Pattarana Sae-Chew, Thidarat Rujirawat, Onrapak Reamtong, Theerapong Krajaejun

**Affiliations:** a Molecular Medicine Program, Multidisciplinary Unit, Faculty of Science, Mahidol University, Bangkok, Thailand; b Research Center, Faculty of Medicine Ramathibodi Hospital, Mahidol University, Bangkok, Thailand; c Department of Molecular Tropical Medicine and Genetics, Faculty of Tropical Medicine, Mahidol University, Bangkok, Thailand; d Department of Pathology, Faculty of Medicine, Ramathibodi Hospital, Mahidol University, Bangkok, Thailand

**Keywords:** *Pythium insidiosum*, Pythiosis, Immunoreactive protein, Protein synthesis, Proteomics

## Abstract

Pythiosis, a severe infectious disease caused by the oomycete *Pythium insidiosum*, continues to cause high levels of morbidity and mortality in humans and animals worldwide. However, there is a need to improve the method for diagnosing and treating the disease, and a better understanding of the causative agent is crucial for such need. In this study, we focused on identifying immunoreactive proteins of *P. insidiosum*, which could serve as promising candidates for diagnostic markers and therapeutic targets. The pathogen crude extract was separated using 2-dimensional gel electrophoresis, and the proteins were analyzed with Western blotting using pythiosis patient sera. Through LC-MS/MS analysis, we identified 55 immunoreactive spots corresponding to 42 unique proteins. Fifteen of these proteins were selected for *in vitro* synthesis, resulting in proteins S01-S21 being generated directly from their PCR-amplified coding sequences. Only 4 synthesized proteins (S01, S11, S12, and S13) exhibited significant immunoreactivity against the pythiosis sera. Among them, S01 provided the highest protein yield and showed promise in differentiating the pythiosis group from the control. Additionally, S01 was annotated as a chaperone DnaK in *P. insidiosum*, part of a protein family involved in host immunity modulation, pathogenesis, and antifungal drug resistance of pathogenic fungi. In summary, we employed an immunoproteomic approach to successfully identify a chaperone DnaK in *P. insidiosum*, which could be a virulence protein of this pathogen. This protein holds potential as a diagnostic marker and therapeutic target for pythiosis and is worth exploring for its clinical application in the future.

## 1 Introduction

*Pythium insidiosum* is a filamentous oomycete organism commonly found in swampy environments, such as stagnant water, reservoirs, and rivers [[1], [2], [3]]. It is the causative agent of “pythiosis,” an infectious disease that threatens humans and animals, especially in tropical and subtropical regions, leading to high morbidity and mortality outcomes [[4], [5], [6], [7], [8], [9]]. This disease primarily presents as cutaneous/subcutaneous, ocular, vascular, and gastrointestinal infections, with the risk of systemic dissemination, presenting clinical challenges in diagnosis and treatment [4,6,10]. Conventional diagnostic methods, relying on culture and histopathological examination, require expertise and are time-consuming, which can delay treatment [6,[11], [12], [13], [14], [15], [16]]. Several immunodiagnostic and molecular-based assays have been established but with limited efficiency or availability [11,[17], [18], [19]]. With no specific drug available for treatment, delayed or inaccurate diagnosis may lead to disease progression into internal vital organs, necessitating prompt and extensive surgery to prevent further infection spread, potentially resulting in disability or death [4,[6], [7], [8],^20^]. Given the rarity of the disease and the lack of practical diagnostic tools, diagnosing pythiosis remains challenging. Hence, there is an urgent need for improved diagnostic and therapeutic strategies for pythiosis.

Two-dimensional (2D) gel electrophoresis is a powerful tool in proteomics used to separate, visualize, and profile complex protein mixtures of a specific pathogen based on their isoelectric points and molecular weights [21]. By analyzing the resulting protein profiles, specific antigens associated with the pathogen's biology, virulence, or immunogenicity can be identified [22,23]. In the case of pythiosis, 2D gel electrophoresis has enabled the identification of immunoreactive proteins from *P. insidiosum* that are recognized by sera from infected individuals [24]. This approach offers a strategy for analyzing the immune response to the pathogen and could provide insights into the interactions with its host, leading to the development of a new disease control method [[24], [25], [26]]. Furthermore, such an immunoproteomic approach could be adopted to identify potential biomarkers for diagnosing pythiosis and monitoring disease progression [24,27,28].

Cell-free protein synthesis (CFPS) is an innovative platform for rapidly and efficiently producing recombinant proteins [29]. By bypassing the need to use living protein-producing host cells, the CFPS system offers flexibility and scalability, making it ideal for *in vitro* synthesizing the proteins of interest [30,31]. Moreover, CFPS allows for incorporating non-standard amino acids, which may be limited in conventional cell-based recombinant protein synthesis, expanding the repertoire of potential antigens [30,32,33]. This study employed an immunoproteomic approach using 2D gel electrophoresis, mass spectrometry coupled with genome-derived proteomic data, CFPS, and immunoassays with the primary aim of identifying, expressing, and characterizing specific immunoreactive proteins of *P. insidiosum*, which are potential candidates for clinical applications in diagnosis and infection control for pythiosis.

## 2 Materials and methods

### 2.1 Microorganism and crude protein preparation

The *P. insidiosum* strain Pi-S, isolated from a patient with pythiosis, was maintained on Sabouraud dextrose (SD) agar at room temperature for 7 days. Ten small agar pieces from the growing organism were transferred to 100 mL of SD broth and incubated with shaking (150 rpm) at 37 °C for 10 days. Hyphae were collected by filtrating the SD broth culture through a 0.22-μm-pore-size Durapore membrane (Merck) before grinding them in a mortar with 10 mL of pre-cooled distilled water. The ruptured hyphae were transferred into a sterile 50 mL conical tube and centrifuged 4000 g at 4 °C for 30 min. The resulting supernatant, now called soluble antigen from broken hyphae (SABH), was measured for protein concentration using Bradford assay [34] and stored at −30 °C until use.

### 2.2 2D gel electrophoresis for separating *P. insidiosum* proteins

Following the previously described method [35], the *P. insidiosum* SABH proteins were separated based on isoelectric points and molecular weights using 2D gel electrophoresis. Briefly, the SABH proteins were cleaned using a 2D-cleanup kit (GE Healthcare Bioscience) before they (100 μg) were loaded onto an IPG strip with a pH range of 3–10 (GE Healthcare Bioscience) and rehydrated overnight in a sample buffer containing 8M urea, 2 % (w/v) 3-[(3-cholamidopropyl) dimethylammonio]-1-propane sulfonate (CHAPS), 15 mM dithiothreitol (DTT), and 0.5 % IPG sample buffer. As the first-dimension protein separation, isoelectric focusing (IEF) was conducted at 300 V for 30 min, 1000 V for 30 min, and 5000 V for 72 min. After that, the IPG strip was equilibrated at 25 °C for 15 min in a 10 mg/mL DTT solution and 25 mg/mL iodoacetamic acid (IAA) solution. Subsequently, the strip was transferred onto a 12 % sodium dodecyl sulfate (SDS) gel for the second-dimension separation based on the protein molecular weights through electrophoresis at 150 V until the guide dye migrated off the gel. The SDS gel was used for Western blot assay against pythiosis sera for immunoreactive protein identification. Identical gels were also stained using silver staining (Sigma-Aldrich) for protein visualization, isolation, and LC-MS/MS-based annotation against the *P. insidiosum*'s proteomic database [36]. Identical gels were also used for Western blot assay against pythiosis sera for immunoreactive protein identification.

### 2.3 Identification of *P. insidiosum* immunogens by Western blot

The 2D separated proteins of *P. insidiosum* (see above) were blotted onto a nitrocellulose membrane using a Power Blotter XL semi-dry transferring machine (Invitrogen) at 1 Amp for 1 h. The protein-blotted membrane was blocked with 5 % skim milk in PBS buffer containing 0.1 % Tween-20 (PBS-T) at room temperature for 1 h and washed once with PBS-T. The blocked membrane was incubated overnight at 4 °C with diluted serum samples (1:2000 in PBS-T with 1 % skim milk) from 3 pythiosis patients and a healthy blood donor, probed with goat anti-human IgG conjugated with horseradish peroxidase (Bio-Rad; 1:10,000 in 1 % skim milk in PBS-T) at room temperature for 2 h, and washed with PBS-T 3 times. After rinsing the processed membrane with PBS-T, protein spots were stained with a chemiluminescence dye (Pierce ECL Western Blotting Substrate; Thermo Fisher) and visualized using a ChemiDocTM MP Imaging System (Bio-Rad).

### 2.4 Annotation of *P. insidiosum* immunogens by LC-MS/MS

The *P. insidiosum* proteins separated in the 2D SDS-PAGE gel were stained with silver staining (Sigma-Aldrich). Only the selected proteins corresponding to the immunoreactive spots identified by Western blot were excised from the gel. Each isolated protein was subjected to trypsin digestion (0.1 mg/mL) and, subsequently, microcapillary liquid chromatography with tandem mass spectrometry (LC-MS/MS) analysis using an Ultimate 3000 nano-LC system (Dionex), following an established protocol [35]. The results of the mass spectrometric data (prepared in ".mgf” file format) were searched against an in-house Mascot proteomic library containing 14,962 predicted proteins from *P. insidiosum* [37].

### 2.5 Cell-free protein synthesis of *P. insidiosum* immunogens

The selected immunoreactive proteins were synthesized using the method outlined by Sae-Chew et al. [38]. Briefly, coding sequences of the immunoreactive proteins identified by LC-MS/MS were retrieved from the *P. insidiosum* genome database [36] and served as a PCR template for designing gene-specific primers by following the Bioneer manufacturer's protocol (Daejeon, Korea). During primer synthesis, an adaptor containing a binding sequence for the second-round PCR primers was added at the 5′-end of forward and revere gene-specific primers. gDNA template (10 ng) extracted from *P. insidiosum* strain Pi-S was amplified in the first-round PCR amplification using the gene-specific primers (Supplementary Table S1), an ExiProgen ProXpress PCR template kit (Bioneer), and the following amplification condition: initial denaturation at 94 °C for 5 min, 30 cycles of denaturation at 94 °C for 30 s, annealing at 58 °C for 30 s, and elongation at 72 °C for 90 s, and final elongation at 72 °C for 5 min. After mixing with 1 μl of the Fluorodye DNA fluorescent loading dye (SMOBIO), the resulting PCR product was separated by 1.5 % agarose gel electrophoresis at 100 V for 30 min and visualized using a BluPAD Dual LED Blue/White light transilluminator (Bio-Helix).

A separated amplicon of interest was then purified using the AccuPrep PCR/Gel Purification kit (Bioneer) and served as a DNA template in the second-round PCR amplification using the ExiProgen ProXpress PCR template kit (Bioneer). Each 20-μl amplification reaction included 10 ng of DNA template (from the first-round PCR), 5 ng of the upstream cassette (containing T7 promoter, ribosome binding site [RBS], and 6-histidine), 5 ng of the downstream cassette (containing the T7 terminator), 10 pmol each of the forward (2F) and reverse (2R) primers (provided in the kit), and nuclease-free water. The amplification condition was set as follows: initial denaturation step at 94 °C for 5 min, 30 cycles of denaturation at 94 °C for 1 min, annealing at 48 °C for 1 min, and elongation at 72 °C for 90 s, and final elongation step at 72 °C for 5 min. The resulting PCR product was checked for its expected size using a QIAxcel Advanced System Capillary Electrophoresis machine (Qiagen), a DNA screening kit with the AM420 method (Qiagen), alignment markers ranging from 15 bp to 5 kb (Qiagen), and a QIAxcel Screen Gel software (Qiagen).

The second-round PCR product was used as a template for CFPS using an ExiProgen EC protein synthesis kit and an ExiProgen automated cell-free protein synthesis machine (Bioneer) for protein expression and purification. Each protein synthesis reaction contained the PCR product (0.5–2 μg), *E. coli* extract, nucleotides, amino acids, and necessary salts and was incubated at 30 °C for 3 h. The expressed protein was purified using affinity purification, which involved the interaction between the 6xHis tag of a target protein and Ni-NTA magnetic nanoparticles at room temperature. The Bio-Rad Protein Assay reagents (Bio-Rad) were used to estimate the protein concentration.

### 2.6 Identifying synthesized proteins by SDS-PAGE and Western blot analysis

Synthesized proteins, alongside the Precision Plus Protein Kaleidoscope pre-stained protein standard (Bio-Rad), which served as a molecular weight marker, were separated by SDS-PAGE (4 % stacking and 12 % separating gel) at 100 V for 70 min, using a Mini-PROTEAN II apparatus (Bio-Rad). The separated proteins in the gel were visualized by staining with Coomassie blue R-250 (Bio-Rad). An identical set of the unstained, separated proteins was transferred onto a nitrocellulose membrane for Western blot analysis as the protocol mentioned above. After the blocking step, the protein-blotted membrane was washed with PBS-T 3 times and incubated with the mouse anti-6xHis monoclonal antibody (Abcam, Cambridge, UK; 1:5000 in PBS-T with 1 % skim milk) at 4 °C overnight before washing again 3 times. The membrane was probed with goat anti-mouse IgG conjugated with horseradish peroxidase (HPR) (Bio-Rad; 1:5000 in 1 % skim milk in PBS-T) at room temperature for 2 h and washed with PBS-T 3 times. The signal on the membrane was developed using a diaminobenzidine tetrahydrochloride (DAB) kit (Thermo Fisher Scientific), and the buffer to DAB substrate ratio was 9:1). After removing the DAB solution, distilled water was added to stop the signal-developing reaction.

For immunoreactive protein detection, the blotted membrane was blocked (5 % skim milk in PBS-T at room temperature for 1 h) and washed three times with PBS-T The membrane was probed with a serum sample (1:2000 in PBS-T with 1 % skim milk at room temperature for 3 h) from a pythiosis patient or a blood donor, and washed three times. The membrane was then incubated with goat anti-human IgG conjugated-HRP (Bio-Rad). A signal was developed using the DAB kit (as above).

### 2.7 Dot blot analysis

Each synthesized protein (1 μl) was placed onto a nitrocellulose membrane and dried overnight. The dotted membrane was blocked with 5 % skim milk in PBS buffer with PBS-T at room temperature for 1 h and washed three times with PBS-T. Next, the blocked membrane was incubated with a serum sample from a pythiosis patient or a blood donor (1:2000 in PBS-T with 1 % skim milk at room temperature for 3 h), washed three times, and probed with goat anti-human IgG conjugated with HRP (Bio-Rad; 1:10,000 in 1 % skim milk in PBS-T) at room temperature for 2 h and washed three times. A signal was developed using the DAB kit as the protocol mentioned above.

### 2.8 Enzyme-linked immunosorbent assay

The ELISA assay, conducted using the previously described procedure [39], involved a series of precise steps. A 96-well flat-bottom polystyrene plate (Corning) was coated with 100 μl/well of a synthesized protein (0.1 μg/mL) in 0.1 M carbonate buffer pH 9.6 [0.2 M Na_2_CO_3_ and 0.2 M NaHCO_3_ (Merck)] and 1.5 % NaCl (Merck) and incubated at 4 °C overnight. The plate was then washed 4 times with the washing buffer [phosphate-buffered saline pH 7.4 (PBS); 137 mM NaCl, 2.7 mM KCl, 10 mM Na_2_HPO_4_, 1.76 mM KH_2_PO_4_ (Merck), and 0.05 % Tween-20 (Calbiochem)]. The plate was blocked with 250 μl of 0.5 % bovine serum albumin (Merck) in PBS at 37 °C for 1 h and washed 4 times.

Duplicate wells of a serum sample (1:1600 in PBS; 100 μl) obtained from human and animal patients with pythiosis (i.e., 18 humans, 4 horses, and 1 dog), as well as individuals without signs of pythiosis (i.e., 15 humans, 5 horses, 2 dogs, a cat, and a cow), were added to each well and incubated at 37 °C for 1 h. The plate was washed with PBS-T and incubated with recombinant protein A/G conjugated with HRP (Thermo Scientific; 1:100,000 in PBS) at 37 °C for 1 h. After another washing step, the ELISA signal was developed using a 3,3′,5,5′-tetramethylbenzidine (TMB) substrate kit (Thermo Scientific) by incubating the ELISA plate at room temperature in the dark for 3 min. The enzymatic reaction was stopped by adding 100 μl/well of 0.5 N sulfuric acid. An optical density (OD) of every sample, including negative control and blank (PBS), was measured at a wavelength of 450 nm. Each blank-subtracted sample OD was divided by that of the negative control serum to generate an ELISA value (EV). The independent *t*-test with a 95 % confidence level was employed to analyze EV variations among serum samples from the pythiosis patients (n = 23) and control individuals (n = 24) using the PASW statistics software version 18 (Statistical Package of Social Sciences Inc., IL, USA).

### 2.9 Bioinformatic analysis

Architectures and conserved domains of an identified immunoreactive protein were predicted utilizing the InterPro software (https://www.ebi.ac.uk/interpro/) and the NCBI Conserved Domain Search Tool (https://www.ncbi.nlm.nih.gov/Structure/cdd/wrpsb.cgi). Additionally, biochemical properties, post-translational modifications, and signal peptide of the subject protein were identified using the programs ScanProsite (https://prosite.expasy.org/scanprosite/) and ProtParam (https://web.expasy.org/protparam/).

## 3 Results

### 3.1 Identification of *P. insidiosum's* immunoreactive proteins

SABH, a crude protein extract from *P. insidiosum* strain Pi-S, was separated using 2D gel electrophoresis. In this process, the first dimension relied on the isoelectric point (pI), and the second dimension depended on protein sizes. After silver staining, we observed many proteins separated in 2 dimensions (pI at pH ranging from 3 to 10 and molecular weights of up to 130 kDa) (Supplementary Fig. S1). These proteins were then blotted onto a nitrocellulose membrane and probed against 3 pythiosis sera [serum IDs: PY066 (Fig. 1A), PY015 (Fig. 1B), and PY035 (Fig. 1C)] and a control serum from a blood donor [serum ID: BB128 (Fig. 1D)]. We found 55 spots that reacted with at least one pythiosis serum but not the blood donor serum (control). The corresponding spots were excised from the silver-stained 2D gel and subjected to protein identification using LC-MS/MS analysis searched against the *P. insidiosum*'s proteomic database [36,37]. This annotation process provided 62 hits corresponding to 42 unique proteins (9 were hypothetical proteins, while the rest can be assigned a function; Table 1). Some spots matched more than one protein, such as spot 16 (i.e., fumarylacetoacetase and 4-hydroxyphenylpyruvate dioxygenase), spot 31 (i.e., serine protease family S33 and heat shock 70-kDa protein), and spot 38 (i.e., enolase, 6-phosphogluconate dehydrogenase, aldehyde dehydrogenase, glycerol kinase, and ATP synthase). On the other hand, some proteins were matched by more than one spot, such as chaperone Dnak (i.e., spots 2 and 3), proteasome subunit alpha type-6 (i.e., spots 21 and 22), ATP synthase subunit beta (i.e., spots 38 and 39), malate dehydrogenase (i.e., spots 35 and 45), AGC protein kinase (i.e., spots 6 and 10), and coatomer subunit beta'-2-like protein (i.e., spots 43 and 44).

**Fig. 1:**
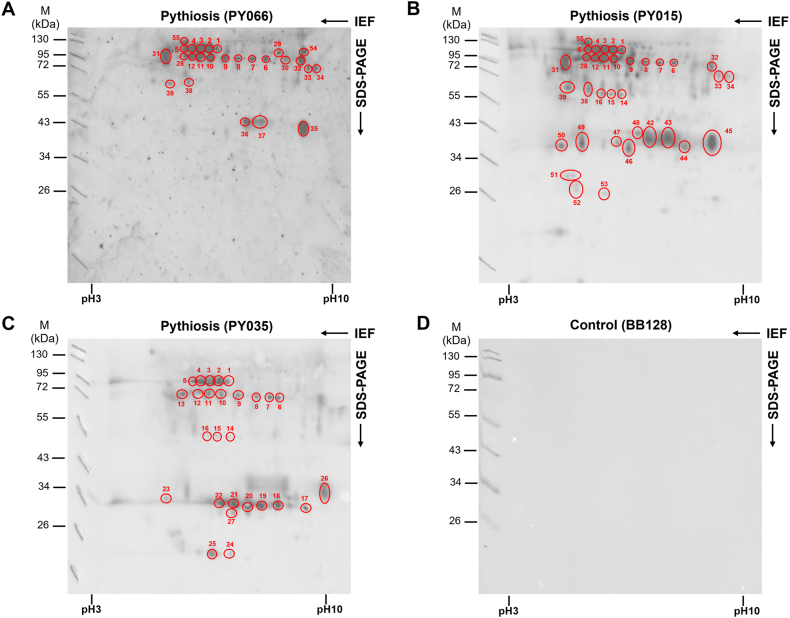
Western blot analysis of SABH, a crude protein extract from *P. insidiosum* strain Pi-S. The proteins are separated in 2 dimensions based on the isoelectric focusing point at pH 3–10 (X-axis) and molecular weights (Y-axis) before probing against 3 pythiosis sera (PY066, PY015, and PY035; **A-C**) and a control serum from a blood donor (BB128; **D**). Western blot signals are visualized using the goat anti-human IgG conjugated with horseradish peroxidase and a chemiluminescence dye substrate. The molecular weight markers (M) are shown on the left, and the pH range is demonstrated at the bottom of each figure. The original and unprocessed images of Fig. 1 are available as supplementary files: Original_Fig1A.tif, Original_Fig1B.tif, Original_Fig1C.tif, and Original_Fig1D.tif.

**Table 1:** A list of immunoreactive spots subjected to the selection for cell-free protein synthesis. 2D gel electrophoresis and Western blot analysis of SABH (*P. insidiosum* crude extract) against 3 pythiosis (PY066, PY015, and PY035) and 1 control (BB128) sera identify 55 immunoreactive spots for protein function annotation by LC-MS/MS searched against our in-house *P. insidiosum* proteome.

Protein spot	Size on 2D gel (kDa)	Protein immunoreactivity by Western blot against patient sera				LC-MS/MS-based protein identification				Protein size difference (%)^b^	Selected protein for CFPS
		PY066	PY015	PY035	BB128	Annotation (COG group)^a^	Accession	Predicted protein size (kDa)	Number of mapped peptides		
1	95	+	+	+	–	Hypothetical Protein (i)	GAX96218.1	168	1	76.8	–
2	95	+	+	+	–	Chaperone Dnak (ii)	GAX92393.1	87	3	8.4	Yes
3	95	+	+	+	–	Chaperone Dnak (ii)	GAX92393.1	87	2	8.4	–
4	95	+	+	+	–	Hypothetical Protein (iv)	GAX96606.1	51	1	46.3	–
5	95	+	+	+	–	Hypothetical Protein (iv)	GAX96606.1	51	1	46.3	–
6	80	+	+	+	–	Agc Protein Kinase (iv)	GAY02450.1	109	1	36.3	–
7	80	+	+	+	–	Hypothetical Protein (iv)	GAX96606.1	51	1	36.3	–
8	80	+	+	+	–	Hypothetical Protein (iii)	GAX97213.1	391	1	388.8	–
9	80	+	+	+	–	Hypothetical Protein (iv)	GAX96554.1	116	1	45.0	–
10	80	+	+	+	–	Agc Protein Kinase (iv)	GAY02450.1	109	1	36.3	–
11	80	+	+	+	–	Hypothetical Protein (iv)	GAY05971.1	42	1	47.5	–
12	80	+	+	+	–	Hypothetical Protein (iv)	GAY05971.1	42	2	47.5	–
13	80	–	–	+	–	Heat Shock 70 Kda Protein, Mitochondrial precursor (ii)	GAX99732.1	70	15	12.5	–
14	48	–	+	+	–	Glycine amidinotransferase (i)	GAY04431.1	46	2	4.2	Yes
15	48	–	+	+	–	Ornithine Aminotransferase, Mitochondrial (iii)	GAX92425.1	48	9	–	Yes
16	48	–	+	+	–	Fumarylacetoacetase (iii)	GAX95036.1	44	3	8.3	Yes
	48	–	+	+	–	4-Hydroxyphenylpyruvate Dioxygenase (iii)	GAX98449.1	48	2	–	Yes
17	28	–	–	+	–	3-Hydroxyacyl-Coa Dehydrogenase, Putative (iv)	GAX93424.1	27	3	3.6	–
18	30	–	–	+	–	Hypothetical Protein (iii)	GAX97213.1	391	1	1203.3	–
19	30	–	–	+	–	Hypothetical Protein (iv)	GAX99991.1	177	1	490.0	–
20	28	–	–	+	–	Hypothetical Protein (iii)	GAX97213.1	391	2	1296.4	–
21	30	–	–	+	–	Proteasome Subunit Alpha Type-6, Putative (ii)	GAX92198.1	27	5	10.0	–
22	30	–	–	+	–	Proteasome Subunit Alpha Type-6, Putative (ii)	GAX92198.1	27	2	10.0	–
23	30	–	–	+	–	Hypothetical Protein (i)	GAX96218.1	28	1	6.7	–
24	22	–	–	+	–	Hypothetical Protein (iv)	GAX96606.1	51	2	131.8	–
25	22	–	–	+	–	Hypothetical Protein (iv)	GAX96606.1	51	2	131.8	–
26	40	–	–	+	–	Ornithine Carbamoyltransferase (iv)	GAX95911.1	39	1	2.5	–
27	27	–	–	+	–	Peroxiredoxin-4 (ii)	GAX94489.1	61	2	125.9	–
28	80	+	+	–	–	Hypothetical Protein (iv)	GAX94634.1	155	1	93.8	–
29	95	+	–	–	–	5-Methyltetrahydropteroyltriglutamate-Homocysteine S-Methyltransferase (iii)	GAX96865.1	87	2	8.4	–
30	80	+	–	–	–	Hypothetical Protein (iv)	GAX96554.1	116	2	45.0	–
31	80	+	+	–	–	Serine Protease Family S33, Putative (iv)	GAX92657.1	211	3	163.8	–
	80	+	+	–	–	Heat Shock 70 Kda Protein (ii)	GAX96752.1	239	19	198.8	–
	80	+	+	–	–	Heat Shock 70 Kda Protein (ii)	GAX99452.1	71	9	11.3	Yes
32	80	+	+	–	–	Hypothetical Protein (iv)	GAX96606.1	51	2	36.3	–
33	64	+	+	–	–	Glycerol-3-Phosphate Dehydrogenase, Mitochondrial Precursor (iii)	GAY03489.1	68	4	6.3	Yes
34	64	+	+	–	–	Glucose-6-Phosphate Isomerase (iii)	GAY02969.1	59	4	7.8	Yes
35	40	+	–	–	–	Malate Dehydrogenase, Nad-Dependent (iii)	GAX96042.1	35	24	12.5	–
36	43	+	–	–	–	Cysteine Synthase A (iv)	GAX99857.1	34	5	20.9	–
37	43	+	–	–	–	Glyceraldehyde-3-Phosphate Dehydrogenase (iii)	GAY03523.1	36	1	16.3	–
38	59	+	+	–	–	Enolase (iii)	GAY02289.1	39	5	33.9	–
	59	+	+	–	–	6-Phosphogluconate Dehydrogenase (iii)	GAX94243.1	53	4	10.2	Yes
	59	+	+	–	–	Aldehyde Dehydrogenase, Putative (iii)	GAX96905.1	52	4	11.9	Yes
	59	+	+	–	–	Glycerol Kinase (iii)	GAY04081.1	56	3	5.1	Yes
	57	+	+	–	–	ATP Synthase Subunit Beta, Mitochondrial (iii)	GAX95749.1	54	15	5.3	Yes
39	57	–	+	–	–	ATP Synthase Subunit Beta, Mitochondrial (iii)	GAX95749.1	54	33	5.3	–
40	63	–	+	–	–	Cytosol Aminopeptidase, Putative (iii)	GAX93219.1	57	9	9.5	–
41	64	–	+	–	–	Aldehyde Dehydrogenase (iii)	GAX92574.1	55	3	14.1	–
42	38	–	+	–	–	Hypothetical Protein (iv)	GAY00607.1	36	20	5.3	–
43	38	–	+	–	–	Coatomer Subunit Beta'-2-Like Protein (iv)	GAY03339.1	36	19	5.3	–
44	34	–	+	–	–	Coatomer Subunit Beta'-2-Like Protein (iv)	GAY03339.1	36	21	5.9	–
45	36	–	+	–	–	Malate Dehydrogenase, Nad-Dependent (iii)	GAX96042.1	35	22	2.8	–
46	36	–	+	–	–	Pyridoxal Biosynthesis Lyase Pdxs (iii)	GAX97134.1	34	1	5.6	–
47	36	–	+	–	–	Hypothetical Protein (iv)	GAY00607.1	36	3	–	–
48	41	–	+	–	–	Hypothetical Protein (iv)	GAY00607.1	36	10	12.2	–
49	36	–	+	–	–	Hypothetical Protein (iv)	GAX94634.1	155	1	330.6	–
50	34	–	+	–	–	Pyruvate Dehydrogenase E1 Component Subunitbeta, Mitochondrial (iii)	GAX93671.1	39	1	14.7	–
51	28	–	+	–	–	Proteasome Subunit Alpha Type-5, Putative (ii)	GAX99254.1	27	1	3.6	–
52	26	–	+	–	–	Proteasome Subunit Beta Type3 Putative (iv)	GAY01217.1	23	1	11.5	–
53	25	–	+	–	–	Peroxiredoxin-2 (ii)	GAY02440.1	22	12	12.0	–
54	100	+	–	–	–	Hypothetical Protein (iii)	GAX96465.1	90	1	10.0	–
55	130	+	+	–	–	Hypothetical Protein (iii)	GAX97213.1	391	1	200.8	–

a The 42 identified proteins are classified into 4 functional groups based on the Clusters of Orthologous Groups (COG): (i) Information storage and processing; (ii) Cellular processes and signaling; (iii) Metabolism; and (iv) Poorly characterized.

b The percent difference in size between a protein spot observed in a 2D gel and its predicted protein size of LC-MSMS-matched protein.

The spots selected for protein expression were based on the following criteria: (i) Identified by at least 2 pythiosis sera using a Western blot assay (for example, spots 3 and 14 were immunoreactive against 3 and 2 screening sera and thus met this criterion, while many other spots showing none or only immunoreactivity against one serum were excluded; Fig. 1); (ii) Mapped by at least 2 peptides through LC-MS/MS analysis (for example, spots 14 and 15 had 2 and 9 mapped peptides, meeting this criterion, while spots 1 and 4 were excluded because only one mapped peptide was detected in the LC-MS/MS analysis; Table 1); and (iii) Showed less than a 15 % difference in size between the protein spot on the 2D gel and its LC-MS/MS-matched protein (for instance, spot 2 was included as it demonstrated a size difference of 8.4 % based on the percent difference of its estimated size on the 2D gel (95 kDa) compared to the predicted size based on the protein sequence (87 kDa), whereas spot 30 was excluded, as it exhibited a size difference of 45.0 %, based on the same calculation; Fig. 1 and Table 1). Following these criteria, 9 spots matching 12 unique proteins were chosen for protein synthesis (Table 1). Spots 2 and 3 were combined for analysis as they had the same molecular size (∼95 kDa) and matched the same protein (Chaperone Dnak; Accession: GAX92393.1). The following additional matched proteins were identified in spots 31 and 38 and included in the downstream protein synthesis step due to their strong Western blot signal or a high number of LC-MS/MS-mapped peptides, despite their predicted size being greater than 15 % different compared to what appeared on the 2D gel. These proteins include heat shock 70-kDa protein (GAX96752.1), serine protease family S33 (GAX92657.1), and enolase (GAY02289.1). In total, 15 matched proteins (as their annotations, accessions, and other characteristics shown in Table 2) were proceeded for CFPS and immunological characterization.

**Table 2:** Cell-free protein synthesis of the selected immunoreactive spots. Coding sequences of 9 selected protein spots are amplified from the genomic DNA of *P. insidiosum* by 2 rounds of PCR amplification to add adaptors necessary for an *in vitro* cell-free protein synthesis (CFPS). CFPS products (full or partial proteins) are checked by SDS-PAGE and Western blot (WB) analyses.

Protein spot	LC-MS/MS-based annotation	Accession	Protein size (kDa)	Gene length (bp)	Number of exons	Protein ID	Expressed portion	Coding sequence (bp)	Calculated protein size (kDa)	First round PCR	Second round PCR	CFPS product		
												SDS PAGE	WB	Size (kDa)
2,3	Chaperone Dnak	GAX92393.1	86.5	2364	1	S01	Full length	2600	87.3	+	+	+	+	90.0
33	Glycerol-3-Phosphate Dehydrogenase	GAY03489.1	68.0	1857	1	S02	Full length	2093	68.8	+	+	+	–	–
34	Glucose-6-Phosphate Isomerase	GAY02969.1	59.1	1629	2	S03	Exon 1	1649	51.4	(−)	ND	ND	ND	ND
14	Glycine Amidinotransferase	GAY04431.1	46.0	1203	3	S04	Exon 1	770	21.0	+	(−)	ND	ND	ND
						S05	Exon 3	821	23.3	+	+	+	+	22.0
15	Ornithine Aminotransferase	GAX92425.1	48.5	1329	5	S06	Exon 4	926	25.6	+	+	+	+	24.0
16	4-Hydroxyphenylpyruvate Dioxygenase	GAX98449.1	47.9	1302	3	S07	Exon 3	596	14.3	+	+	–	–	–
	Fumarylacetoacetase	GAX95036.1	43.6	1200	6	S08	Exon 3	638	15.6	+	+	–	–	–
						S09	Exon 6	584	13.3	+	+	+	+	13.0
31	Heat Shock 70 kDa Protein	GAX96752.1	239.0	6474	21	S10	Exon 8	632	15.0	+	+	–	–	–
						S11	Exon 9	1424	43.8	+	+	+	+	50.0
						S12	Exon 19	1808	58.6	+	+	+	–	–
	Heat Shock 70 kDa Protein	GAX99452.1	71.1	1965	0	S13	Full length	2201	71.9	+	+	+	+	70.0
	Serine Protease Family S33	GAX92657.1	210.7	5808	4	S14	Exon 4	2228	72.7	+	+	–	–	–
38	ATP Synthase Subunit Beta	GAX95749.1	53.6	1509	2	S15	Exon 1	545	11.8	+	+	+	–	–
						S16	Exon 2	1142	43.5	+	+	+	+	45.0
	Aldehyde Dehydrogenase	GAX96905.1	52.0	1452	10	S17	Exon 9	653	15.5	+	+	+	–	–
	Enolase	GAY02289.1	39.5	1092	0	S18	Full length	1328	40.3	+	+	+	+	40.0
	Glycerol Kinase	GAY04081.1	55.7	1530	0	S19	Full length	1766	56.5	+	+	+	+	60.0
	6-Phosphogluconate Dehydrogenase	GAX94243.1	52.6	1449	2	S20	Exon 1	1157	33.6	+	+	+	+	35.0
						S21	Exon 2	770	20.9	+	+	+	–	–

### 3.2 Cell-free synthesis of *P. insidiosum's* proteins

For primer design, the coding sequences of 15 selected proteins were retrieved from the *P. insidiosum* strain Pi-S genome database [36]. Since the coding sequence used in the CFPS process is limited to a maximum length of ∼2000 bp for the use of an amplicon as a protein synthesis template, only 5 proteins (assigned as protein IDs S01, S02, S13, S18, and S19; Table 2) could be synthesized in full-length. The remaining 10 proteins can undergo partial protein synthesis, giving rise to up to 3 expressed portions per protein, depending on the gene size, number of exons, and peptide mapping regions. For instance, the heat shock 70 kDa protein (GAX96752.1) was synthesized in three portions, using exon 8 (assigned as protein S10), exon 9 (protein S11), and exon 19 (protein S12). Fumarylacetoacetase (GAX95036.1) was produced in two portions, using exon 3 (protein S08) and exon 6 (protein S09). Ornithine aminotransferase (GAX92425.1) was only generated in one protein, using exon 4 (protein S06). The characteristics of all 21 proteins (S01-S21) subjected to CFPS reaction are shown in Table 2.

A total of 21 gene-specific primer pairs were designed and synthesized (Supplementary Table S1). These primers contained an adaptor with a binding sequence for the second-round PCR primers (2F and 2R) at the 5′-end of the forward and reverse gene-specific primers. They were used in the first-round PCR to amplify 5 full-length and 16 partial protein-coding sequences from the gDNA of *P. insidiosum* strain Pi-S. The expected proteins were denoted as protein IDs S01- S21 (Table 2). The first-round PCR amplified the expected amplicon size for 20 out of 21 target coding sequences. The resulting amplicons were then used as a template for the second-round PCR, utilizing primers 2F and 2R. During the amplification process, the upstream cassette containing the T7 promoter, RBS, and 6-histidine, as well as the downstream cassette containing the T7 terminator, were added to each target coding sequence, as these components are essential for protein synthesis. All the coding sequences were successfully amplified except those of the S03 and S04 proteins (Table 2). Nineteen resulting PCR products were purified and sequenced to ensure the accuracy of the target coding sequences before proceeding to the CFPS step.

Up to 16 different proteins can be synthesized simultaneously using a second-round PCR product as a template for protein synthesis and a commercial CFPS kit from Bioneer with an automated protein synthesis machine from the same company. The positive control for protein synthesis was the green fluorescent protein (GFP) coding sequence. The entire process (i.e., transcribing and translating each coding sequence and purifying the resulting product into a ready-to-use protein using affinity-based purification) took 6 h. Following synthesis, all the proteins were separated using SDS-PAGE, transferred onto a nitrocellulose membrane, and underwent Western blot analysis using a mouse anti-6x-His tag antibody to confirm the presence of the expected proteins. The SDS-PAGE gel stained with Coomassie Blue revealed several prominent background proteins at 75, 40, and 25 kDa from the *E. coli* extract in all samples (Fig. 2A). An expected protein band was observed in the CFPS reactions of 15 out of 19 target coding sequences (all except S07, S08, S10, and S14), as shown in Fig. 2A and Table 2. Further analysis using Western blot showed that 10 coding sequences were successfully translated into a protein with the expected size (i.e., S01, S05, S06, S09, S11, S13, S16, S18, S19, and S20) that was reacted with the mouse anti-6x-His tag antibody (Fig. 2B and Table 2). Among them, proteins S06, S18, and S20 showed a faint Western blot signal.

**Fig. 2:**
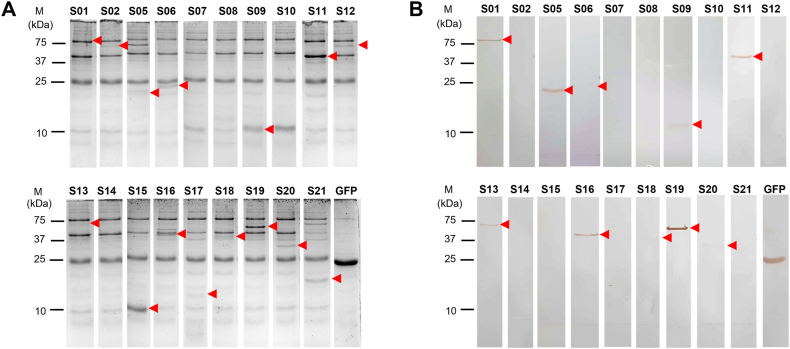
SDS-PAGE and Western blot analysis of *P. insidiosum*'s synthesized proteins. Nineteen coding sequences of the recruited target proteins S01-S21 (except S03 and S04) serve as a protein synthesis template in the CFPS reaction, and the resulting products are checked for their presence using SDS-PAGE stained with Coomassie blue dye (**A**) and their immunoreactivity using Western blot analysis probed against the mouse anti-6x-His tag antibody (**B**). Green fluorescent protein (GFP) is a positive control for the CFPS reaction. An arrowhead indicates a target synthesized protein. The molecular weight markers (M) are shown on the left of each figure. The original unprocessed images of Fig. 2 are available as supplementary files: Original_Fig2A_S01-02_S11-S14_GFP.tif, Original_Fig2A_S05-06.tif, Original_Fig2A_S07-10.tif, Original_Fig2A_S015-21.tif, Original_Fig2B_S01-S02_S11-S14_GFP.tif, Original_Fig2B_S05-06.tif, Original_Fig2B_S07-10.tif, and Original_Fig2B_S015-21.tif.

### 3.3 Immunoreactivity of synthesized *P. insidiosum* proteins

In Western blot analysis, only 10 of 19 CFPS reactions resulted in the expected protein products detected using the mouse anti-6x-His tag antibody (Table 2). However, protein products from all 19 CFPS reactions were assessed for immunoreactivity using dot blot analysis and patient sera (Fig. 3A). The dot blot panel included crude extracts from *P. insidiosum* (SABH) and *E. coli* as control antigens. The protein products and the crude extracts were probed against 3 pythiosis sera (IDs: PY066, PY015, and PY035) and a control serum from a blood donor (ID: BB128). The control serum displayed no or faint background signal with all proteins in the panel. All protein products from CFPS and the 2 crude extracts showed a noticeable signal against the same pythiosis serum (PY066). Proteins S12 and S13 exhibited marked dot-blot reactivity with pythiosis sera PY066 and PY035, while proteins S01 and S11 showed a strong signal for all three pythiosis sera. The *E. coli* crude extract yielded an above-background signal for 2 out of 3 pythiosis sera (PY066 and PY015).

**Fig. 3:**
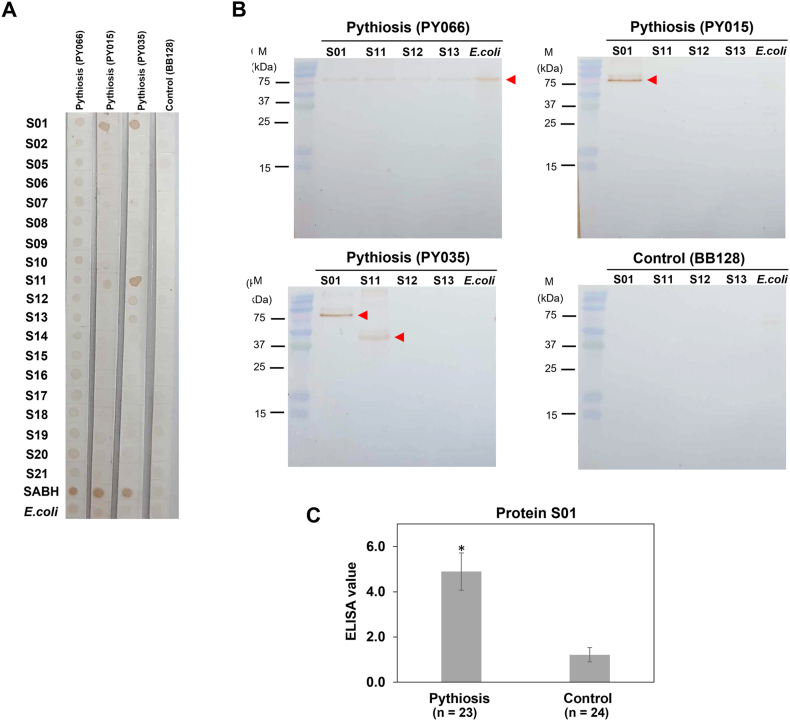
Immunoreactivity assessment of the synthesized proteins from *P. insidiosum*. All 19 CFPS-derived proteins of *P. insidiosum* (S01 – S21, except S03 and S04) are evaluated for their immunoreactivity against 3 pythiosis sera (i.e., PY066, PY015, and PY035) and a control serum from a blood donor (BB128) using dot blot (**A**) and Western blot (**B**) analyses. The crude extracts from *P. insidiosum* (SABH) and *E. coli* are control antigens. An arrowhead indicates an immunoreactive band. The molecular weight markers (M) are shown on the left of the Western blot membrane. The protein S01 is further evaluated for its immunoreactivity against an extensive set of pythiosis (from 18 humans, 4 horses, and 1 dog) and control (from 15 humans, 5 horses, 2 dogs, a cat, and a cow) serum samples using ELISA (**C**). An asterisk shows a statistical significance (p-value <0.05). The original unprocessed images of Fig. 3 are available as supplementary files: Original_Fig3A.tif, Original_Fig3B_PY066.tif, Original_Fig3B_PY015.tif, Original_Fig3B_PY035.tif, and Original_Fig3B_BB128.tif.

The proteins S01, S11, S12, and S13, which reacted with at least two pythiosis sera, underwent further Western blot analysis to confirm their sizes and immunoreactivity (Fig. 3B). This analysis used *E. coli* crude extract as control antigens to check for cross-reactivity. The control serum (BB128) did not react to any protein, while the pythiosis serum PY066 notably stained the same band (falling between 75 and 100 kDa) of all proteins tested. All three pythiosis sera consistently reacted to a compatible molecular weight of the protein S01 (∼90 kDa). Protein S11 showed a Western blot signal at its expected size (44 kDa) only with the pythiosis serum PY035. Proteins S12 and S13 did not show reactivity at their calculated sizes (59 and 72 kDa) when tested against all pythiosis sera.

Out of all CFPS products, only the protein S01 showed immunoreactivity at its estimated molecular size (∼90 kDa), as confirmed by dot blot and Western blot analyses probed against the pythiosis sera (Fig. 3A and B). In a subsequent experiment, the immunogenicity of protein S01 was further investigated using ELISA with a more extensive set of pythiosis (from 18 humans, 4 horses, and 1 dog) and control (from 15 humans, 5 horses, 2 dogs, a cat, and a cow) serum samples (Fig. 3C). The protein S01 was coated in a 96-well ELISA plate and tested against these 23 pythiosis and 24 control serum samples. The average ELISA value obtained from all pythiosis sera (mean: 4.89; standard error of the mean (SEM): 0.83) was significantly higher than that from the control sera (mean: 1.21; SEM: 0.32) (p-value <0.05).

### 3.4 Predicted architecture of the protein S01

The genomic [36] and transcriptomic [40] data of *P. insidiosum* revealed that the coding sequence of the protein S01 comprises 2364 bases, translating to a polypeptide of 787 amino-acid long (Fig. 4). LC-MS/MS analysis mapped 4 peptides into the protein S01 at the amino acid positions: 244–259, 301–314, 360–381, and 731–739 (Fig. 4A). This protein was predicted to have a molecular weight of 86.5 kDa, contain no signal peptide, and include 2 conserve domains: Suppressor of G2 allele of SKP1 (SGT1; amino acid positions: 30 to 129) and Heat Shock 70-kDa Protein (Hsp70; amino acid positions: 189 to 782) (Fig. 4C). The protein S01 was also predicted to possess various post-translational modifications, including 11 sites of protein kinase C phosphorylation, 11 sites of N-myristoylation, 7 sites of N-glycosylation, 18 sites of casein kinase II phosphorylation, and 1 site of cAMP/cGMP-dependent protein kinase phosphorylations (Fig. 4B). Additionally, the chaperone DnaK sequences from *P. insidiosum* (accession: GAX92393.1; this study) were compared with those from *Cryptococcus neoformans* (accession: XP_012052733.1) and *Aspergillus terreus* (accession: XP_001209480.1). All these proteins contained one heat shock protein (Hsp70) domain, which ranges in size from 594 to 604 amino acids (Fig. 4C, D, and 4E). The Hsp70 domains exhibited sequence similarity ranging from 76 % to 79 %.

**Fig. 4:**
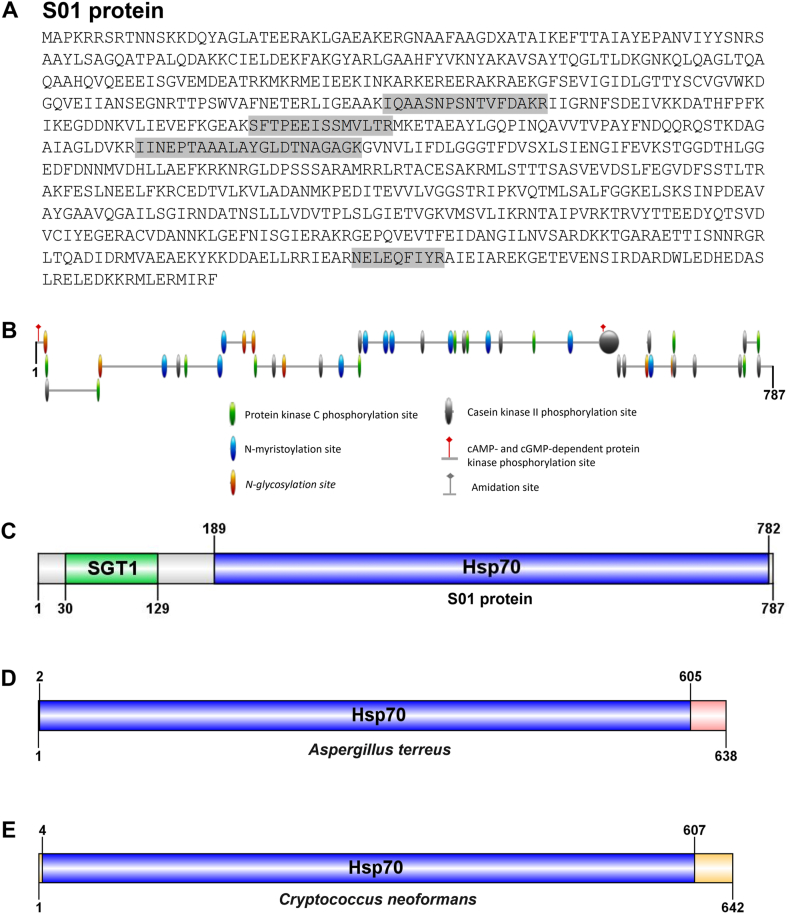
The architecture of the *P. insidiosum* protein S01. LC-MS/MS analysis can map peptides (labeled gray) at 4 different regions (amino acid positions 244–259, 301–314, 360–381, and 731–739) in the 787 amino acid-long S01protein (accession: GAX92393.1) (**A**). The protein S01 contains various predicted post-translational modifications, including protein kinase C phosphorylation, N-myristoylation, N-glycosylation, casein kinase II phosphorylation, and cAMP/cGMP-dependent protein kinase phosphorylations (**B**). Two conserved domains, namely Suppressor of G2 allele of SKP1 (SGT1; amino acid positions: 30 to 129) and Heat shock 70-kDa protein (Hsp70; amino acid positions: 189 to 782), are predicted in the S01 protein (**C**). The homologous chaperone DnaK proteins, containing a Hsp70 domain, from *Aspergillus terreus* (accession: XP_001209480.1) (**D**) and *Cryptococcus neoformans* (accession: XP_012052733.1) (**E**) are depicted for comparison with the S01 protein.

## 4 Discussion

The study involved separating the crude extract proteins of the reference *P. insidiosum* strain Pi-S (which has complete genome data) into two dimensions (2D) based on pI and molecular weights to enhance the resolution for identifying and isolating target proteins as candidate biomarkers. The entire 2D process is complex and time-consuming compared with one-dimensional separation, which relies solely on protein sizes. Additionally, this procedure can only analyze one sample at a time, limiting its use in simultaneously studying and comparing multiple proteins. After staining a 2D gel with a silver dye, hundreds of separated pathogen proteins were observed. Representative sera from 3 pythiosis patients (who were infected with different *P. insidiosum* strains likely expressing the same proteins but with some amino acid variations) contained overlapping and distinct sets of antibodies against proteins from the *P. insidiosum* strain Pi-S (Fig. 1) and identified 55 immunoreactive protein spots, corresponding to 42 unique proteins (defined by LC-MS/MS; Table 1). Some proteins showed immunoreactivity across all 3 serum samples, while others did not. This experimental approach provided findings that underscore the variability and consistency in immunoreactivity and host immune responses of *P. insidiosum* proteins. The 42 identified proteins can be categorized into 4 functional groups based on the Clusters of Orthologous Groups (COG) [41]: (i) Information storage and processing (2 proteins; 4.8 %); (ii) Cellular processes and signaling (8 proteins; 19.0 %); (iii) Metabolism (18 proteins; 42.9 %); and (iv) Poorly characterized (14 proteins; 33.3 %). The immunoreactive proteins were narrowed down to a more practical set through stringent protein selection criteria regarding immunoreactivity, peptide mapping, and protein length. As an outcome, a handful of 15 immunoreactive proteins can be recruited for downstream protein synthesis (Table 2).

In a comparable 2D gel-based Western blot analysis, Chechi et al. identified 23 immunoreactive proteins using pooled sera from humans and horses [24]. In contrast, our study found 42 immunoreactive proteins using individual serum samples from 3 patients with pythiosis. Most of the proteins reported by Chechi et al. did not overlap with those identified in our study, suggesting that differences in the serum sample sets may influence the results of the two studies. Additionally, discrepancies in the tools utilized for LC-MS/MS, the proteomic databases, *P. insidiosum* strains, and protein extraction methods can significantly affect the outcomes of the analyses. For instance, different models or machines for LC-MS/MS may yield varying results due to the impact of their mechanical and software settings on the analysis, even when the same protein sample is examined. Chechi et al. used the SwissProt database (taxonomy: *Phytophthora infestans*) [24], while our study relied on a *P. insidiosum*-specific proteomic database for LC-MS/MS-based protein identification. This difference can affect both the identification of proteins and their functional annotation. Nonetheless, a few immunoreactive proteins we identified had annotations that matched proteins reported by Chechi et al., including 6-phosphogluconate dehydrogenase, heat shock 70-kDa protein, and glycerol-3-phosphate dehydrogenase. To address the need for validation of the identified immunoreactive proteins, we expressed all 15 selected proteins using the CFPS platform and confirmed their immunoreactivity through dot blot, Western blot, and ELISA analyses against pythiosis sera.

The full-length coding sequences of 15 recruited proteins were obtained from the *P. insidiosum* genome database [37]. Due to size limitations and the presence of exons, only 5 proteins with coding sequences less than 2000 bp were fully synthesized, while each of the rest was produced in up to 3 partial segments, resulting in a total of 21 proteins being generated (S01-S21; Table 2). Fifteen of 21 proteins (71.4 %) were successfully translated, and 10 (47.6 %) were properly expressed as they reacted with the mouse anti-6xHis tag antibody. Failure to produce 11 proteins (52.4 %) occurred at the pre-protein synthesis step (target sequences not amplified; n = 2), the protein synthesis step (target proteins not translated; n = 4), and the post-protein synthesis step (target proteins not in proper structure; n = 5). Regarding production yield, 3 proteins (S06, S18, and S20) were expressed relatively low compared to others, quantitatively limiting their use in downstream applications. Furthermore, the presence of prominent background proteins (at 75, 40, and 25-kDa) from the *E. coli* extract in the CFPS kit could interfere with detecting and analyzing the synthesized protein, underscoring the need to improve the protein purification step. Despite its limitations, CFPS offers a straightforward and rapid process, making it suitable for the simultaneous production of multiple proteins in this study, a task that is difficult to accomplish using conventional methods for synthesizing recombinant proteins.

*P. insidiosum* crude proteins (SABH), *E. coli* extract, and all 19 CFPS reactions were initially screened for immunoreactivity using dot blot analysis probed with 3 sera (PY066, PY015, and PY035) from different pythiosis patients (Fig. 3A). The sera from the individual patients displayed distinct immunoreactivity patterns across all CFPS proteins. This variability is likely due to the unique and overlapping sets of antibodies produced by patients against different strains of *P. insidiosum*. These individual differences in pythiosis sera were significant for identifying common immunoreactive CFPS proteins. Consequently, only 4 CFPS proteins (S01, S11, S12, and S13) were recognized by at least 2 of the pythiosis sera. This highlights that some *E. coli*-synthesized proteins of a eukaryotic organism like *P. insidiosum* may lack the proper protein structure and post-translational modifications necessary for effective antibody binding.

Notably, the pythiosis serum PY066 reacted with the *E. coli* extract, SABH, and all CFPS samples (containing *E. coli* antigens from the CFPS kit; Fig. 2A), suggesting that this serum contained both anti-*E. coli* and anti-*P. insidiosum* antibodies, so interpreting the protein immunoreactivity observed with the serum is not feasible. Western blot analysis was then used to visualize and confirm the molecular size of each immunoreactive protein (Fig. 3B). The results showed that only the protein S01 consistently displayed immunoreactivity with all 3 pythiosis sera at the expected size (∼90 kDa). It is worth noting that the pythiosis serum PY066 reacted with a band of equivalent size to protein S01 across all the samples (including that of the *E. coli* extract), making it challenging to distinguish true from the cross-reactive signal for this protein. Despite this, S01 demonstrated the best yield and immunoreactivity among all synthesized proteins. The diagnostic capability of the *P. insidiosum* protein S01 was evaluated using ELISA with a larger sample set from both pythiosis patients (n = 23) and control individuals (n = 24) (Fig. 3C). The results revealed that the average ELISA value for the pythiosis serum samples was significantly higher (4 folds) than that of the controls (4.89 vs 1.21). In other words, this protein can distinguish between the pythiosis and control groups, suggesting a robust host anti-S01 antibody response in the presence of *P. insidiosum* infection. The homologous S01 protein is present across multiple strains of *P. insidiosum*, as shown by a BLAST search of the protein sequence against the NCBI protein database (https://blast.ncbi.nlm.nih.gov/Blast.cgi). Furthermore, S01 was identified as a chaperone DnaK protein containing the SGT1 and Hsp70 domains (accession: GAX92393.1; Fig. 4C). The function of the homologous chaperone DnaK of *P. insidiosum* (S01 protein) is currently unknown.

Based on COG functional group classification, a significant portion of the identified immunoreactive proteins (18 of 42 proteins; 42.9 %) is involved in metabolic processes (Table 1). Among these proteins, S01 is the only immunoreactive protein capable of distinguishing pythiosis sera from control samples and has been identified as a chaperone DnaK protein. Chaperones are a diverse group of proteins that play critical roles in the folding, assembling, and maintaining other proteins within cells [42]. In pathogenic bacteria, chaperones like DnaK are vital for survival under challenging conditions, including antibiotic exposure and host immune responses [[43], [44], [45], [46]]. By stabilizing proteins essential for bacterial virulence and resistance, these chaperones enable pathogens to grow and cause disease. For instance, the chaperone in *Mycobacterium smegmatis* (a model organism for studying *Mycobacterium tuberculosis*) involves microbial tolerance to drug-induced stress [43]. In *Staphylococcus aureus*, deleting the DnaK gene increases susceptibility to oxidative stress and cell wall-targeting antibiotics, resulting in decreased survival in mouse models [46]. In pathogenic fungi such as *A. terreus* and *C. neoformans*, chaperones are also crucial for disease progression, including evading host immunity, properly folding virulence factors, and developing antifungal resistance [47,48]. Therefore, developing a specific inhibitor targeting chaperone DnaK is a promising strategy for combating many pathogens, particularly drug-resistant strains. However, the role of chaperone DnaK in *P. insidiosum* remains unexplored. This study compared the homologous chaperone DnaK sequences from *P. insidiosum* with those from *C. neoformans* [47] and *A. terreus* [48]. All these proteins share the Hsp70 domain, which ranges in size from 594 to 604 amino acids (Fig. 4C, D, and 4E). Notably, the Hsp70 domains exhibit a significant level of sequence similarity (76–79 %), suggesting that these chaperone DnaK proteins may share similar functions. Notably, the chaperone DnaK of *P. insidiosum* uniquely includes an additional small domain, SGT1, located at the N-terminus (Fig. 4C). Future studies on the detailed structure and function of chaperone DnaK in *P. insidiosum* could provide valuable insights into their roles in disease mechanisms, paving the way for developing diagnostic and therapeutic modalities for pythiosis.

In order to improve future experimental design, it is important to address several limitations associated with CFPS that affect the identification, yield, and purity of immunoreactive proteins from *P. insidiosum* in this study. The CFPS platform employs a prokaryotic protein expression system, which lacks post-translational modifications. As a result, the immunoreactivity of the expressed proteins from the eukaryotic organism *P. insidiosum* is likely diminished. A eukaryotic protein expression system using yeast or mammalian host cells [[49], [50], [51]] could facilitate proper protein folding and post-translational modifications (i.e., disulfide bond formation, glycosylation, phosphorylation, and acetylation) and improve the immunoreactivity of *P. insidiosum* proteins. Additionally, the CFPS platform can only translate protein-coding sequences of up to 2000 bp, which restricts the full expression of larger proteins. While CFPS can simultaneously produce multiple proteins (up to 16 per batch in this study), it yields a relatively low amount of expressed proteins (up to 100 μg per reaction), sufficient only for small-scale experiments or screening. A conventional protein expression system using live host cells (such as bacteria, yeasts, or mammalian cells) is necessary for larger-scale protein synthesis for extensive downstream analysis or applications. Another drawback of CFPS is the low purity of the expressed proteins due to host cell (*E. coli*) protein contamination, which can lead to interference in subsequent analyses, such as immunological cross-reactivity when probing the expressed proteins with a patient serum that contains anti-*E. coli* antibodies, as illustrated in Fig. 2, Fig. 3B. This highlights the need for improvements in the protein purification process by incorporating additional methods beyond affinity purification.

In conclusion, our comprehensive immunoproteomic approach, employing 2D gel electrophoresis, mass spectrometry, in-house proteomic data, CFPS, and immunoassays, identified 42 unique immunoreactive proteins in *P. insidiosum*. Stringent selection criteria reduced the list to 15 immunoreactive proteins, which were more manageable for downstream protein synthesis and characterization. Their retrievable coding sequences led to the *in vitro* synthesis of proteins S01-S21. Only 4 synthesized proteins (S01, S11, S12, and S13) exhibited significant immunoreactivity against pythiosis sera. S01 provided the highest protein yield and ability to differentiate between pythiosis and control groups. Additionally, S01 was functionally annotated as a chaperone DnaK in *P. insidiosum*, with possible implications in host immunity modulation, pathogenesis, and antifungal drug resistance. Our findings suggest that S01 could be a virulence protein of *P. insidiosum* and exhibits the potential for diagnostic and therapeutic applications for pythiosis.

## CRediT authorship contribution statement

**Chalisa Jaturapaktrarak:** Writing – review & editing, Writing – original draft, Visualization, Methodology, Funding acquisition. **Pattarana Sae-Chew:** Writing – review & editing, Resources, Methodology. **Thidarat Rujirawat:** Writing – review & editing, Resources, Methodology. **Onrapak Reamtong:** Writing – review & editing, Resources, Methodology. **Theerapong Krajaejun:** Writing – review & editing, Writing – original draft, Resources, Methodology, Funding acquisition, Conceptualization.

## Informed consent statement

Not applicable.

## Ethical consideration

This work was approved by the Human Research Ethics Committee, Faculty of Medicine Ramathibodi Hospital, Mahidol University (approval number: MURA2023/891) on November 21, 2023.

## Data availability statement

The genome sequences of *P. insidiosum* strains Pi-S are retrievable from the DDBJ/NCBI databases through the accession numbers BBXB01000001–1192. The coding sequences of the identified immunoreactive proteins of *P. insidiosum* can be retrieved using the accession numbers shown in Table 1, Table 2 The mass spectrometry raw data used in this study have been deposited in the Science Data Bank repository under the accession number 10.57760/sciencedb.11360 (https://www.scidb.cn/en/anonymous/cWEyMm1t).

## Declaration of generative AI and AI-assisted technologies in the writing process

During the preparation of this work, the authors used the Grammarly software to enhance the grammar and improve the readability. After using this tool, the authors reviewed and edited the content as needed and take full responsibility for the content of the publication.

## Funding

This work was supported by the 10.13039/501100012309Royal Golden Jubilee Ph.D. Scholarship Program, 10.13039/501100004704National Research Council of Thailand, Thailand (Grant number: PHD/0059/2561; C.J.), 10.13039/501100004704National Research Council of Thailand and 10.13039/501100004156Mahidol University, Thailand (Grant numbers: N42A650339; T.K.) and 10.13039/501100010804Faculty of Medicine, Ramathibodi Hospital, 10.13039/501100004156Mahidol University, Thailand (Grant number: CF_67004; T.K.).

## Declaration of competing interest

The authors declare the following financial interests/personal relationships which may be considered as potential competing interests: Theerapong Krajaejun reports financial support was provided by 10.13039/501100004704National Research Council of Thailand. If there are other authors, they declare that they have no known competing financial interests or personal relationships that could have appeared to influence the work reported in this paper.

## Figures and Tables

**figs1:**
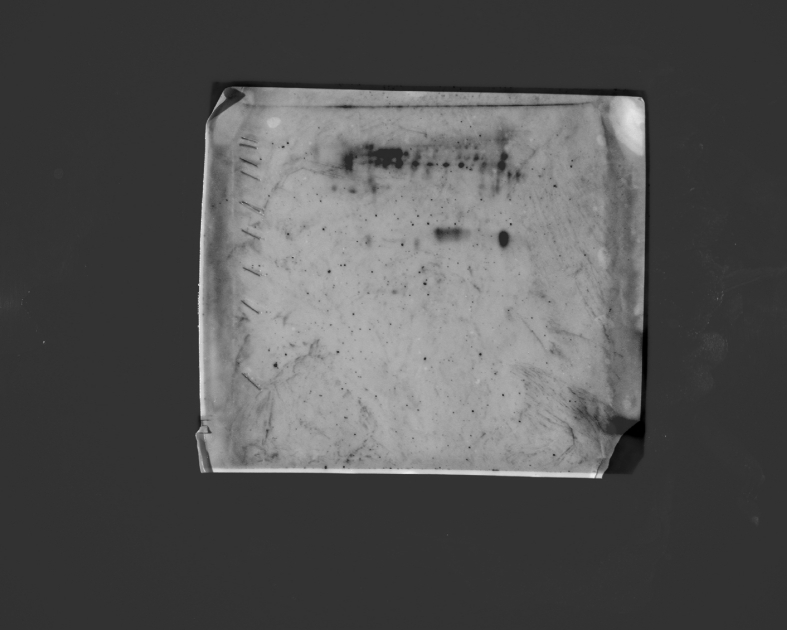


**figs2:**
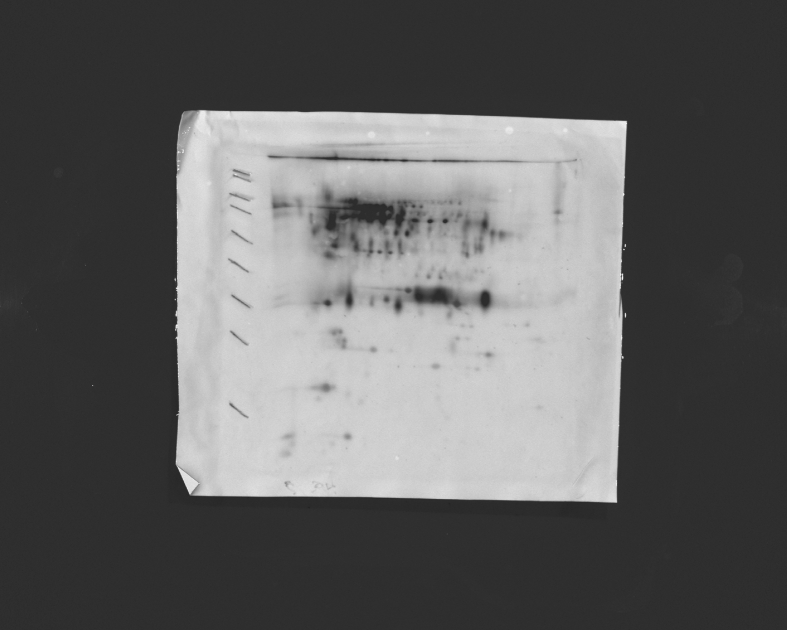


**figs3:**
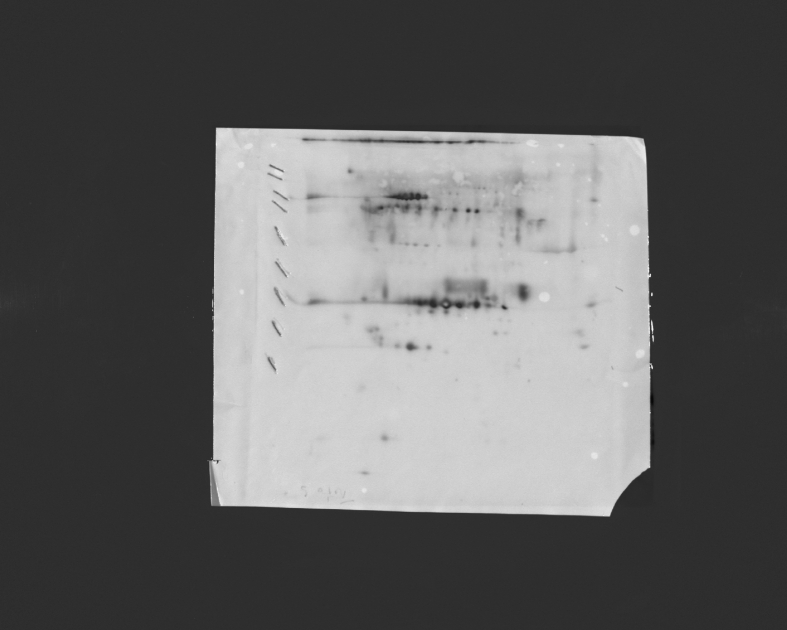


**figs4:**
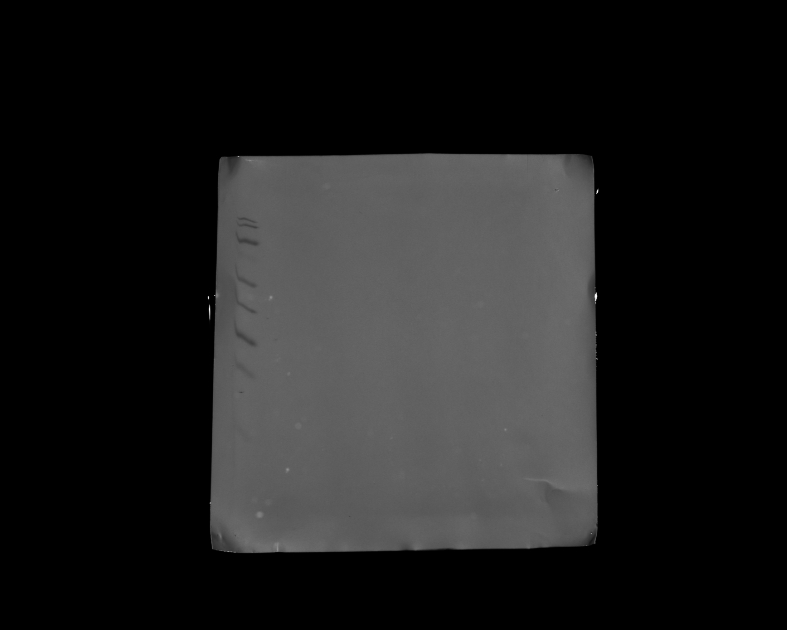


**figs5:**
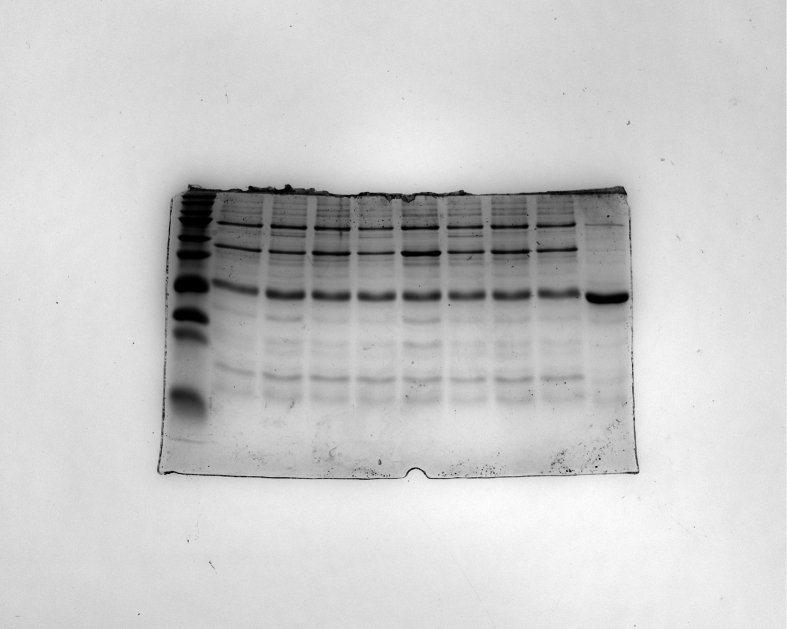


**figs6:**
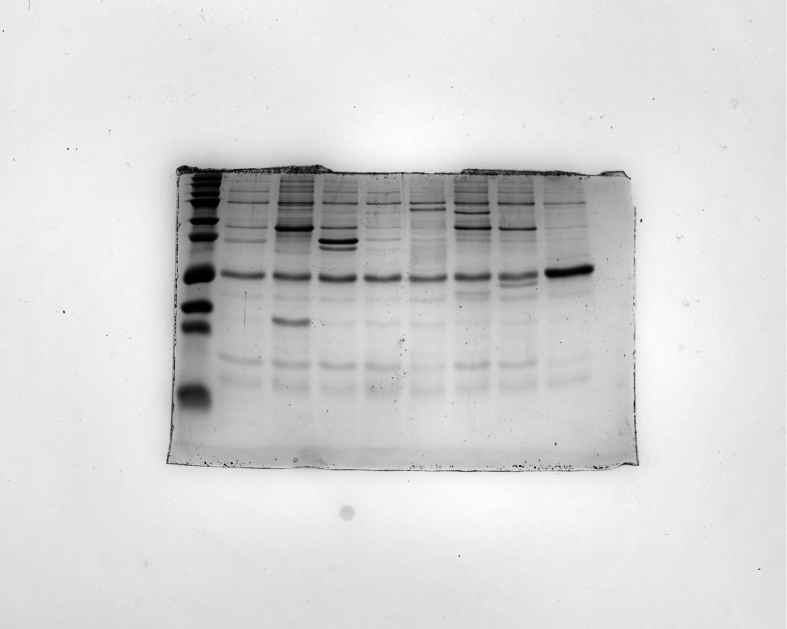


**figs7:**
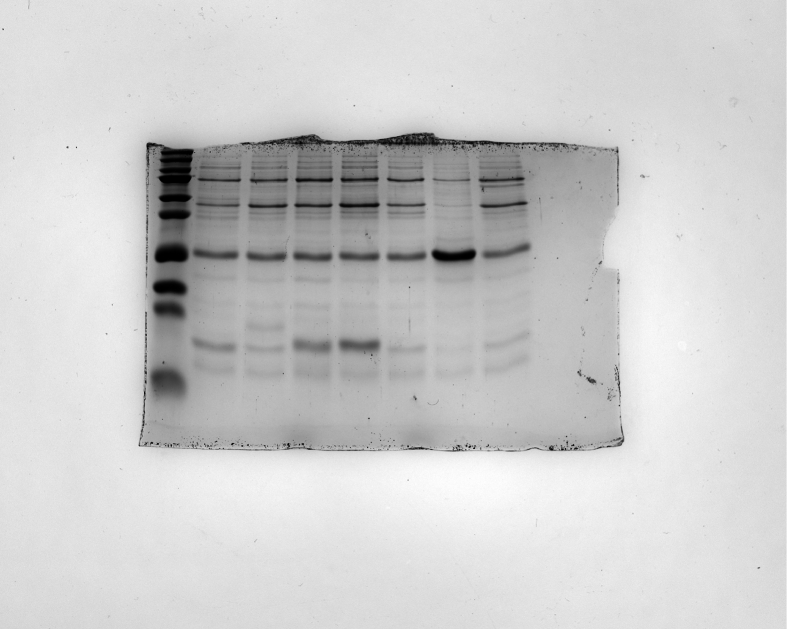


**figs8:**
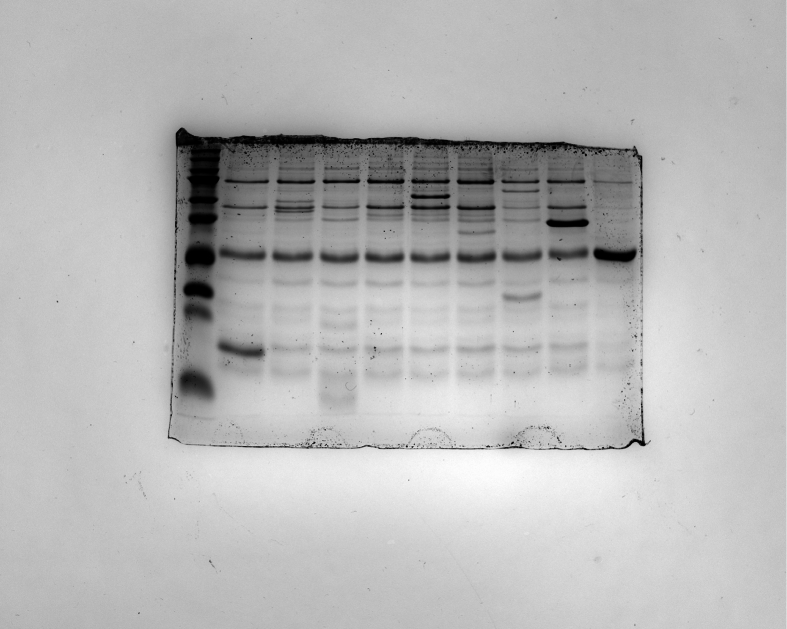


**figs9:**
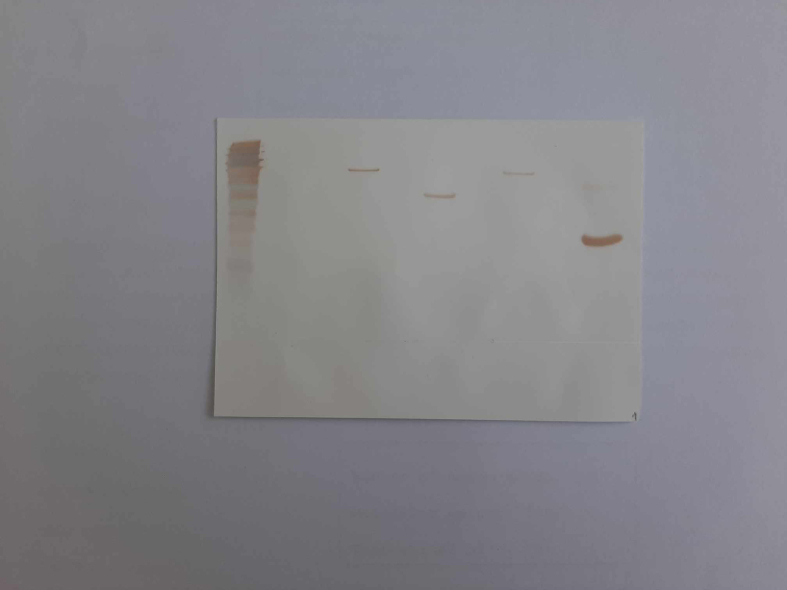


**figs10:**
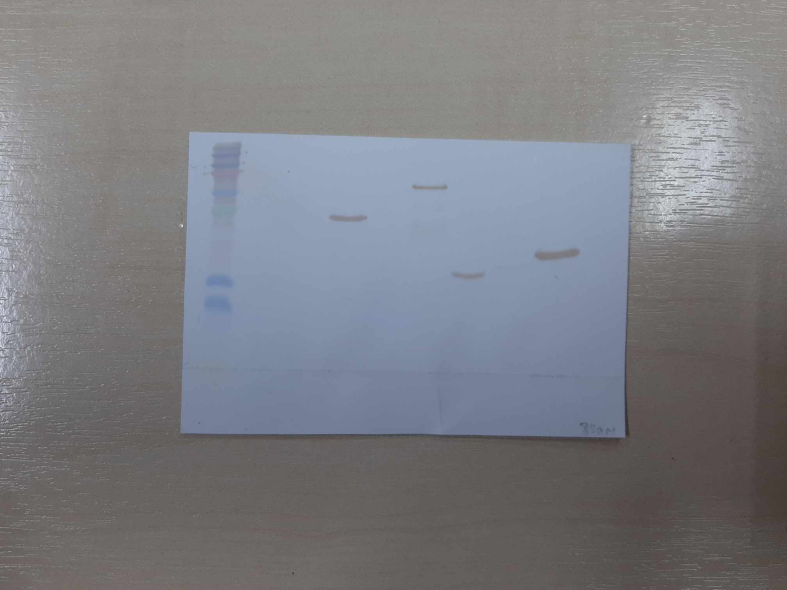


**figs11:**
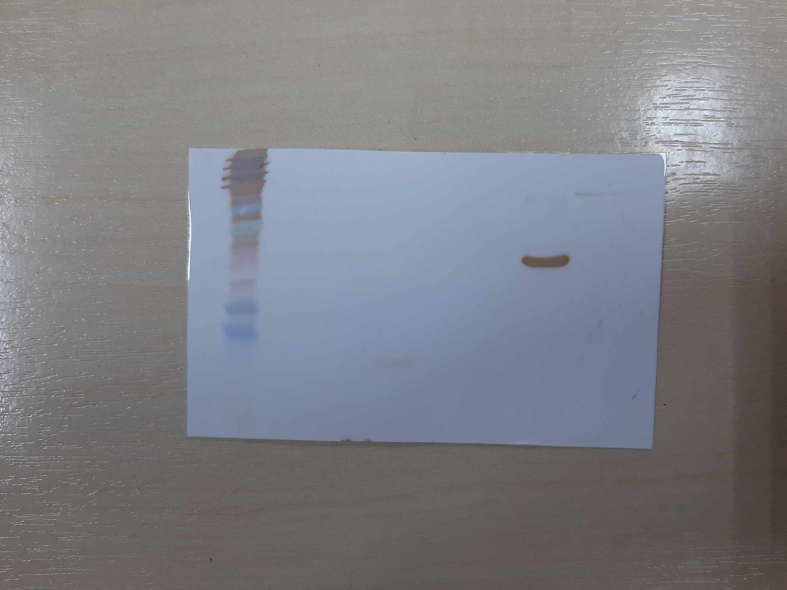


**figs12:**
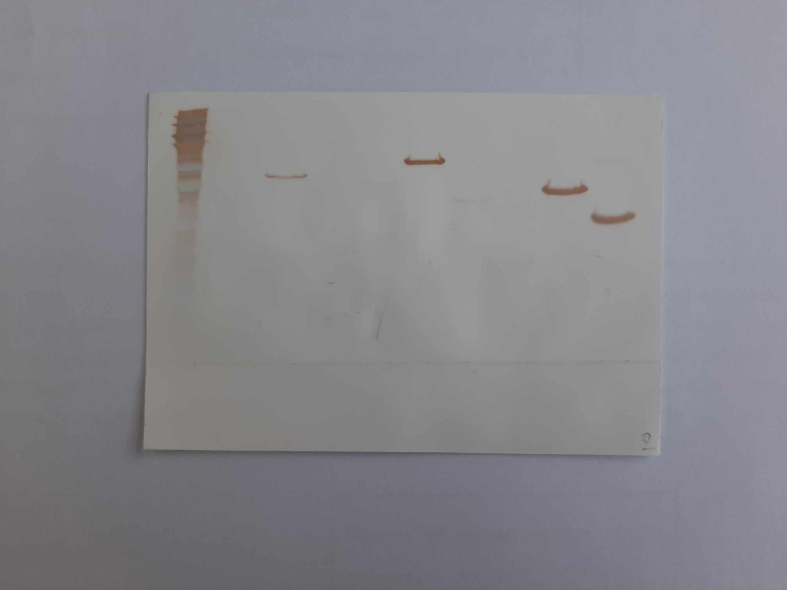


**figs13:**
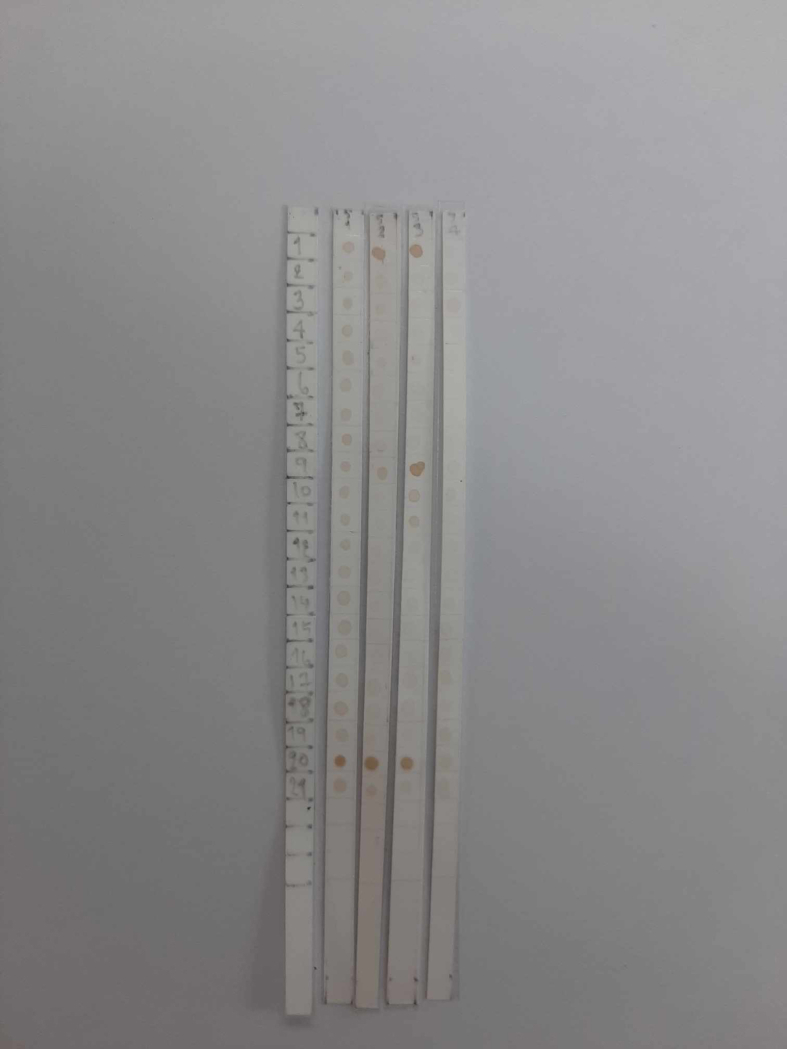


**figs14:**
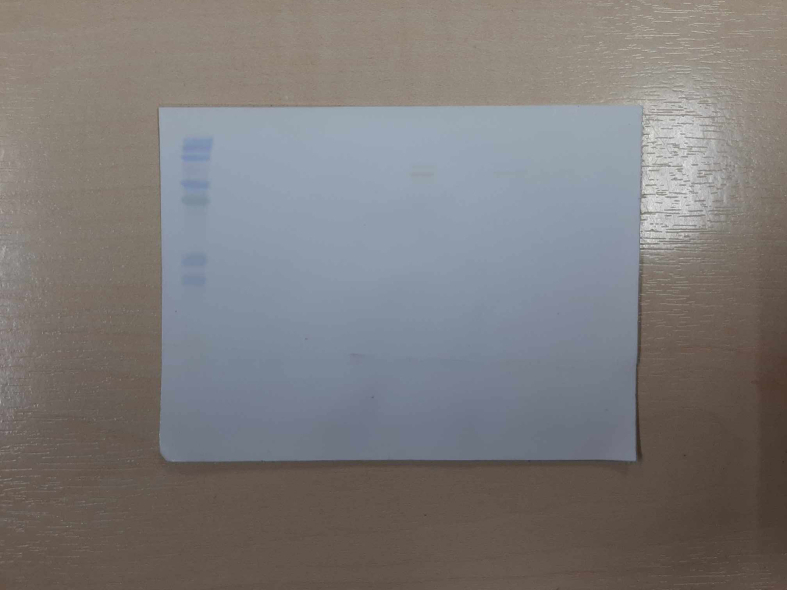


**figs15:**
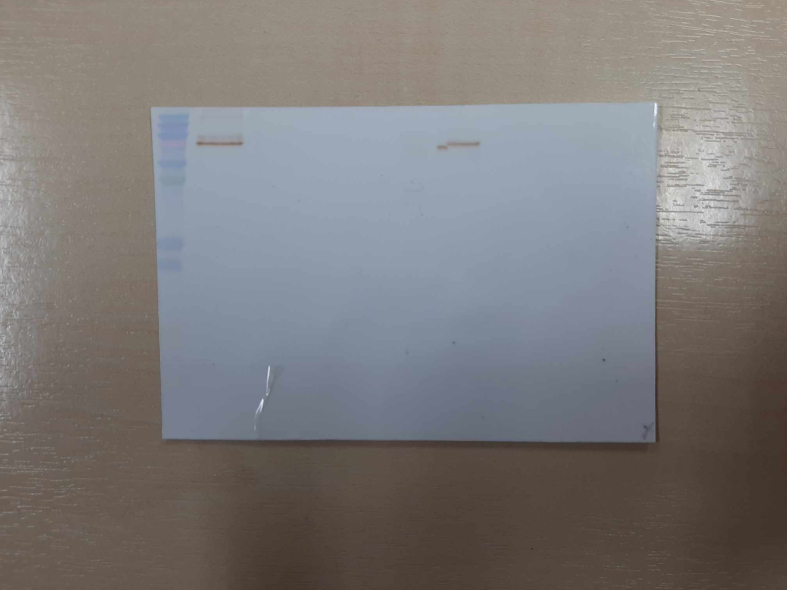


**figs16:**
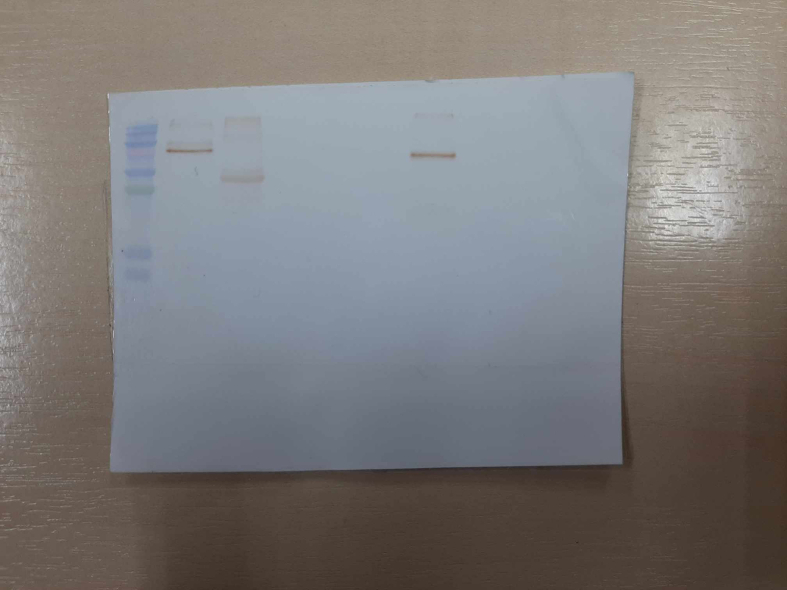


**figs17:**
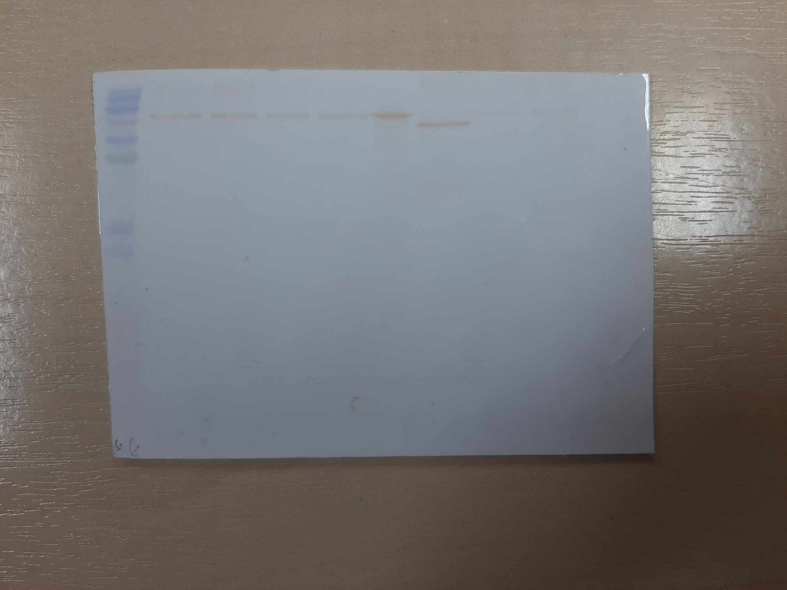

