# Supplementary material for: Immunoproteomic approach identifies a putative virulence chaperone DnaK protein as a candidate diagnostic marker and therapeutic target for Pythium insidiosum infection
Source: Heliyon. 2025 Feb 6;11(4):e42487. doi: 10.1016/j.heliyon.2025.e42487 (PMC11959653; doi:10.1016/j.heliyon.2025.e42487)
Supplement: Multimedia component 1 [file mmc1.pdf]

Supplementary data

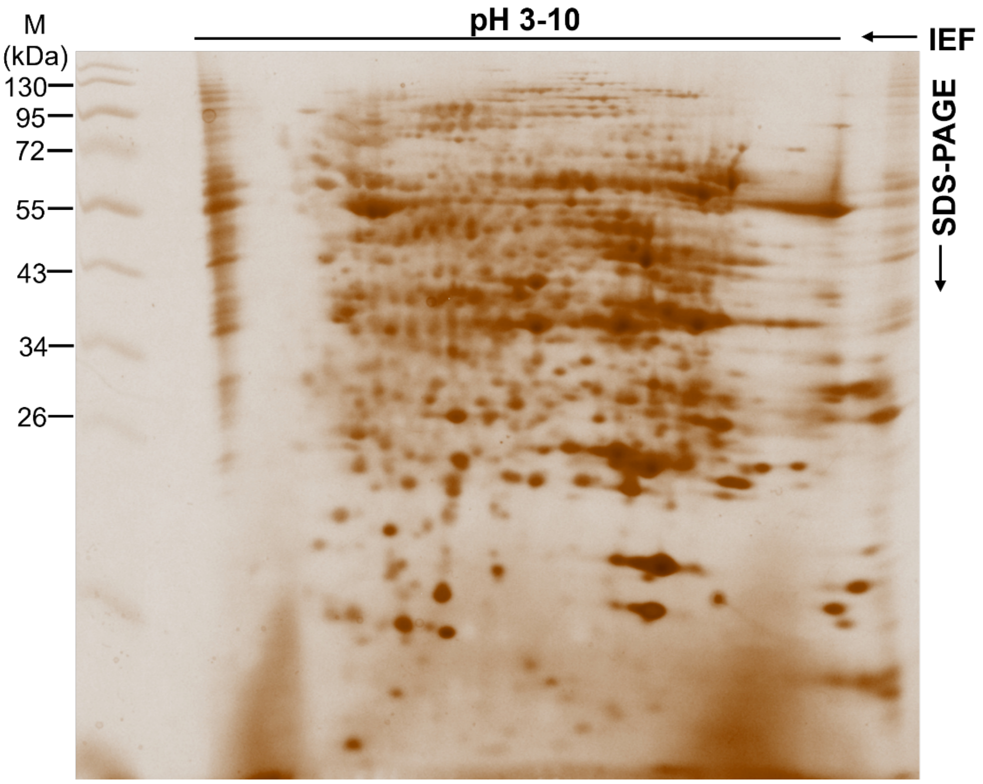

Supplementary Figure S1. An image of 2-dimensional gel electrophoresis of SABH, a crude protein extract from *P. insidiosum* strain Pi-S. The proteins are separated in the first dimension (X-axis), which relies on the isoelectric focusing point (pI) at pH ranging from 3 to 10. In contrast, the second dimension (Y-axis) depends on protein molecular weights through 12% SDS-PAGE gel. The separated proteins are stained with the silver dye. The molecular weight markers (M) are shown on the left, and the pH range is demonstrated at the top.

Supplementary Table S1. Gene-specific primers for the first-round PCR amplification of 21 coding sequences as a part of the cell-free synthesis of *P. insidiosum* proteins (S01-S21).

Protein IDs	Forward primers	Reverse primers
S01	5'-Adaptor-GACGAGGCCACGCGCAAG-3'	5'-Adaptor-GAAGCGGATCATGCGCTC-3'
S02	5'-Adaptor-GCGTCCTCCATGTTCCGT-3'	5'-Adaptor-GTTGTGCATCGTGGCCAG-3'
S03	5'-Adaptor-CCGCTCATCAACGAGACC-3'	5'-Adaptor-GGGAAGAGCTTGTGCGTG-3'
S04	5'-Adaptor-CAGCTCCGCGCTCTCCGT-3'	5'-Adaptor-CTGGAAGTACTCCTTGAT-3'
S05	5'-Adaptor-TCGGTGATCACCGAGTTT-3'	5'-Adaptor-CTGGAGCGTGCCCTTGCG-3'
S06	5'-Adaptor-ATCCTCCCGATGAACACG-3'	5'-Adaptor-GTTCATCTCCGCGCGGAA-3'
S07	5'-Adaptor-GAGATGCGCAAGCGCAGC-3'	5'-Adaptor-CACGTTGAGCGTCTTCTC-3'
S08	5'-Adaptor-GGCAGCAAGGCATGGGCC-3'	5'-Adaptor-CACTTCAAGCTCATAGTC-3'
S09	5'-Adaptor-GACGACGCCAACAACTCG-3'	5'-Adaptor-GATCGGATGCGCCGGCAG-3'
S10	5'-Adaptor-CACTGGCCTTTCACGGTG-3'	5'-Adaptor-CTCGAAGATACCCTCCTC-3'
S11	5'-Adaptor-CTCACGAGCAACCAGCGT-3'	5'-Adaptor-CTCCTCGATCTTCGGGCC-3'
S12	5'-Adaptor-CACTGGCCGTTCCGCGTC-3'	5'-Adaptor-GCGCTGCATGATGGGATT-3'
S13	5'-Adaptor-ACAGCAGTCTCTGGTGCA-3'	5'-Adaptor-GTCCACTTCCTCGATCTT-3'
S14	5'-Adaptor-CTCCTCCCGCGCCGCCTG-3'	5'-Adaptor-AACCTCGCCGTCAGCCGA-3'
S15	5'-Adaptor-TTCTCGCGTGTGGGTCTC-3'	5'-Adaptor-CATGATCGGGTTGCCGGT-3'
S16	5'-Adaptor-GTCCCTGTCGGTCCGGAG-3'	5'-Adaptor-CTCGTCTAATTCGGCCGC-3'
S17	5'-Adaptor-GTCCAGGATGCGATTGCG-3'	5'-Adaptor-CGACTTCAGCTTCGTCAA-3'
S18	5'-Adaptor-GTTGAGACGCTCGACGGC-3'	5'-Adaptor-GAACGACTTGTACTTGCC-3'
S19	5'-Adaptor-GCTACGGAACAATACGTC-3'	5'-Adaptor-CGCCGACTTATCATCCTC-3'
S20	5'-Adaptor-TCGGGGCTCTCGGACGTC-3'	5'-Adaptor-CTTGCTCGCGACCACGCG-3'
S21	5'-Adaptor-ATCCCGGCGGTCGACAAG-3'	5'-Adaptor-GAGGTTGCCACGCACGCG-3'
